# The South American radiation of *Jerrybuccinum* (Gastropoda, Buccinidae), with a new deep-water species from Chile

**DOI:** 10.3897/zookeys.409.7194

**Published:** 2014-05-15

**Authors:** Koen Fraussen, Javier Sellanes, Peter Stahlschmidt

**Affiliations:** 1 Leuvensestraat 25, B-3200 Aarschot, Belgium; 2 Universidad Católica del Norte, Facultad de Ciencias del Mar, Departamento de Biología Marina, Larrondo 1281. Coquimbo, Chile; 3 Institute for Environmental Sciences, University Koblenz-Landau, Fortstrasse 7, D-76829 Landau, Germany

**Keywords:** New taxa, East Pacific, methane seep, low oxygen environments

## Abstract

A new deep water species from off the Chilean coast, *Jerrybuccinum kantori* **sp. n.**, is described. The animal is equipped with a large statocyst. *Kryptos explorator* Fraussen & Sellanes, 2008 from off Concepción is found to be congeneric and transferred to the genus *Jerrybuccinum*. Differences in size and sculpture serve to distinguish the new species from *J. explorator*. Both Chilean species are associated with methane seep or low oxygen environments. They are compared with *J. malvinense* Kantor & Pastorino, 2009 and two still unnamed species from the Falkland Plateau.

## Introduction

The Patagonian marine environments of the SW Atlantic host a vast marine fauna with high pelagic and benthic biomass. The benthic molluscan fauna has been proven to be rich in endemic species and genera. Recent investigations have resulted in the descriptions of a number of genera that are typical of the Patagonian continental slope fauna (e.g. Harasewych et al. 2000, Kantor and Pastorino 2009, Harasewych and Pastorino 2010). The fauna off southern Chile, the Pacific Ocean counterpart of the Patagonian region, is still mostly underestimated by malacologists even though a number of endemic species have recently been described (Fraussen and Hadorn 2000, Holmes et al. 2005, Oliver and Sellanes 2005, Sellanes and Krylova 2005, Houart and Sellanes 2006, Vilvens and Sellanes 2006, Fraussen and Sellanes 2007, Fraussen et al. 2012, Araya 2013, Krylova et al. 2014). For a brief overview of past scientific expeditions that produced noteworthy malacological contributions we refer to Fraussen et al. (2008).

The goals of the present paper are to contribute to the knowledge of the family Buccinidae from north to south-central Chile, comparing species with their SW Atlantic counterparts, and to continue the effort of describing the malacofauna, whether endemic or not, of Chilean methane seeps.

The Concepción Methane Seep Area (CMSA; ~37°S; ~800 m water depth) has been the source of many new species of molluscs in the previous decade. We herein add a new species of gastropod, inhabiting both the CMSA and a shallower water area located northwards. The new area is situated within the lower boundary of the SE Pacific permanent oxygen minimum zone (OMZ; Wyrtki 1962). At this place, the presence of chemosymbiotic bivalves (including a new genus of vesicomyid clam; Krylova et al. 2014), and tubeworms typical of seep communities may indicate the existence of a methane seep habitat, or at least a reducing habitat associated to the OMZ, but further thorough studies are needed to prove this thesis. This area has been named El Quisco Seep Site (after the adjacent coastal town) and it is located at about 350 m depth at approximately 33°S.

The new species is compared with its two congeneric species known so far, one from the Atlantic and one from the Pacific, as well as with two still undescribed species from the Patagonian shelf.

## Materials and methods

The material of the new species described in this paper was collected during the following cruises: AIW (R/V Vidal Gormáz), ONR (R/V Vidal Gormáz) and INSPIRE (R/V Melville).

For radula preparation the body of paratype 1 was extracted from the shell and dissected to isolate the buccal complex. It was treated with a 1% solution of sodium hypochlorite until the soft tissue was completely dissolved. Subsequently, the radula was cleaned in several shifts of distilled water, unfolded and mounted for SEM examination.

### Abbreviations

AGT Agassiz trawl

CBUCN Colecciones Biológicas Universidad Católica del Norte, Coquimbo, Chile

CMSA Concepción Methane Seep Area

EQSS El Quisco Seep Site

KBIN Koninklijk Belgisch Instituut voor Natuurwetenschappen, Brussels, Belgium

KF collection of Koen Fraussen, Belgium

MNHN Muséum national d’Histoire naturelle, Paris, France

MNHNCL Museo Nacional de Historia Natural, Santiago, Chile

PS collection of Peter Stahlschmidt, Germany

lv live collected specimen

dd empty shell

## Results

### Systematics
Class Gastropoda Cuvier, 1797
Order Neogastropoda Wenz, 1938
Superfamily Buccinoidea Rafinesque, 1815
Family Buccinidae Rafinesque, 1815

#### Genus Jerrybuccinum

Kantor & Pastorino, 2009

http://species-id.net/wiki/Jerrybuccinum

##### Type species.

*Jerrybuccinum malvinense* Kantor & Pastorino, 2009 (type locality: Falkland Islands (Islas Malvinas), 52°00'S, 56°36'W, R/V Eltanin cruise 7 st 558, 384–494 m).

##### Diagnosis.

*Jerrybuccinum* is characterised by a slender, fusiform shell with a high spire but a moderately long siphonal canal, a broad but blunt protoconch ornamented with fine spiral cords, a sculpture consisting of rather short, slightly bended axial ribs on the adapical part of the whorls and two or more accentuated spiral cords at the transition to the base. In most species (*Jerrybuccinum malvinense*, the type species, *Jerrybuccinum explorator* and *Jerrybuccinum* species 1) the spiral cord delimitating the base forms a strong keel. This characteristic is subject to variation within the genus as the keel may be replaced by one or more weaker but slightly broader spiral cords (*Jerrybuccinum kantori* sp. n. and *Jerrybuccinum* species 2).

The radula is characterised by a rectangular central tooth with slightly curved base and with one (Kantor and Pastorino 2009) or two (present paper) small cusps and tricuspid lateral teeth with a long basal projection at the outer side.

*Kryptos* Jeffreys in Dautzenberg and Fischer 1896 (type species *Kryptos elegans*, Jeffreys in Dautzenberg and Fischer 1896 = *Pleurotomella koehleri* Locard, 1896) differs by the presence of axial sculpture on the protoconch (rather than only fine spiral cords), a broad interspace situated between the spiral cords along the periphery (rather than a spiral keel), carinated whorls (type species) or sculptured with sharp keels along the periphery (*Kryptos tholoides* (Watson, 1882)), an operculum with terminal nucleus (rather than just falling within the margin) and a radula with a rectangular middle tooth without prominent cusps (Bouchet and Warén 1985: fig. 487). Bouchet and Warén (1985: 196) noted that *Kryptos koehleri* lacks eyes.

*Americominella* Klappenbach & Ureta, 1972 (type species: *Americominella duartei* Klappenbach & Ureta, 1972, a senior synonym of *Americominella longisetosus* (Castellanos & Fernandez, 1972)) (= *Echinosipho* Kaiser, 1977, type species: *Echinosipho aculeatum* Kaiser, 1977 a senior synonym of *Americominella duartei*) from the Patagonian continental shelf is somewhat similar in protoconch morphology but differs by the radula, which has a clearly tricuspid central tooth with longer cusps and a broader base.

*Buccipagoda* Ponder, 2010 (type species: *Kapala kengrahami* Ponder, 1982) from Australia has a radula with an identical central tooth, but which differs by the lateral teeth with one large outer cusp and more than 5 small inner cusps.

*Antarctoneptunea* Dell, 1972 (type species: *Fusitriton aurora* Hedley, 1916) from off Antarctica is similar in shape but differs in the absence of axial sculpture, in having a large papilliform protoconch, a radula with a tricuspid central tooth and a much larger adult size.

*Prosipho* Thiele, 1912 (type species: *Prosipho gaussianus* Thiele, 1912) is a rather heterogeneous group (Engl 2012) mainly living near Antarctica, with slender shells ornamented with a dominant spiral sculpture. Some *Jerrybuccinum*, especially the smaller species without obvious axial sculpture like *Jerrybuccinum* species 2, may be confused with them. *Prosipho* differs by the smaller protoconch with smooth whorls, the axial sculpture that is usually absent or consisting of fine and sharp minute ridges that are straight (rather than broad and inclined ribs) and different radula morphology.

##### Included species.

*Jerrybuccinum malvinense* Kantor & Pastorino, 2009 (Falkland Plateau), type species;

*Jerrybuccinum explorator* new combination (Fraussen and Sellanes 2008) (Chile);

*Jerrybuccinum kantori* new species (Chile);

*Jerrybuccinum* species 1 (Falkland Plateau);

*Jerrybuccinum* species 2 (Falkland Plateau).

#### Jerrybuccinum malvinense

Kantor & Pastorino, 2009

http://species-id.net/wiki/Jerrybuccinum_malvinense

Figures 15–16 , 27

Jerrybuccinum malvinense Kantor & Pastorino, 2009: 49-52, figs 1–12.

##### Type material.

Holotype in USNM-887765. Paratype in USNM-898774.

**Figures 1–11.**
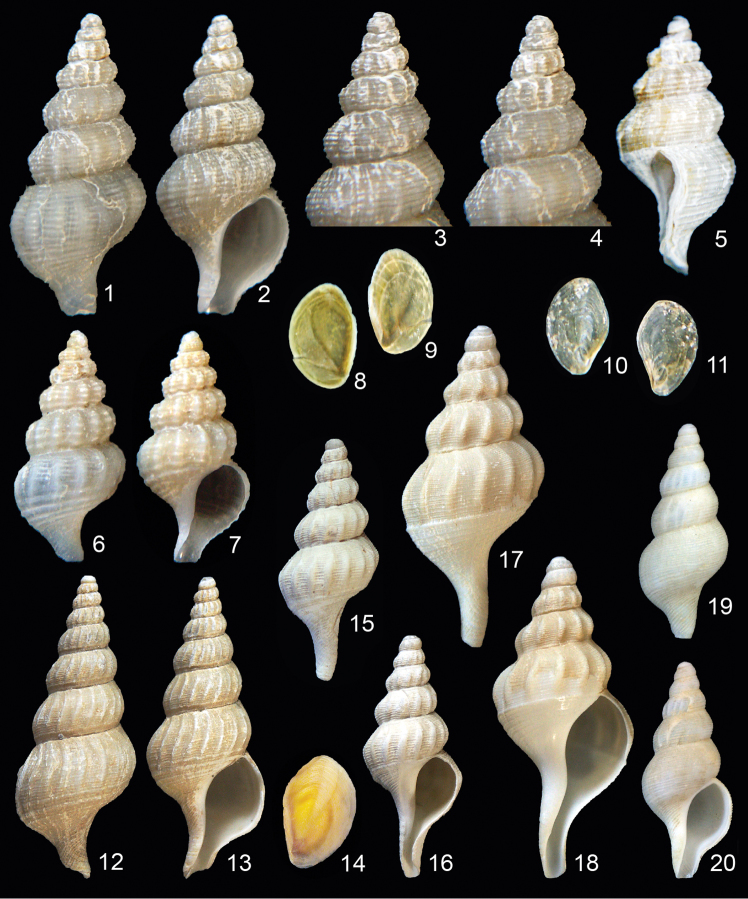
*Jerrybuccinum kantori* sp. n. **1–4** Holotype, 14.5 mm, Chile, northwest of the Bay of Concepción, R/V Melville, INSPIRE cruise, AGT 04, 36°23.595'S, 73°42.910'W, ~700 m, MNHNCL-7589 **5** Paratype 7, 12.5 mm, Chile, off El Quisco, R/V Melville, INSPIRE cruise, AGT 10, 33°23.378'S, 71°52.782'W, ~340 m, PS-150148 **6–7** paratype 9, 9.5 mm, same locality as paratype 7, MNHNCL-7592 **8–9** operculum of paratype 6, 4.2 mm **10–11** operculum of paratype 9, 2.5 mm **12–14** *Jerrybuccinum explorator* (Fraussen & Sellanes, 2008) **12–13** Paratype 3, 28.9 mm, Chile, off Concepción, 36°22'68 S, 73°42'46 W, 708–709 m, KF-5180 **14** Operculum of holotype, 6.6 mm, Chile, northwest of the Bay of Concepción, 36°20'97 S, 73°44'86 W, 850 m, MNHNCL-5866 **15–16** *Jerrybuccinum malvinense* Kantor & Pastorino, 2009 1, 19.6 mm, Falkland Plateau, 700 m, KF-1989 **17–18** *Jerrybuccinum* species 1, 34.4 mm, Falkland Plateau, 700 m, KF-1609 **19–20** *Jerrybuccinum* species 2, 18.2 mm, north off Falkland Islands, 51°S, 60°W, 850–900 m, KF-1763.

**Figures 21–27.**
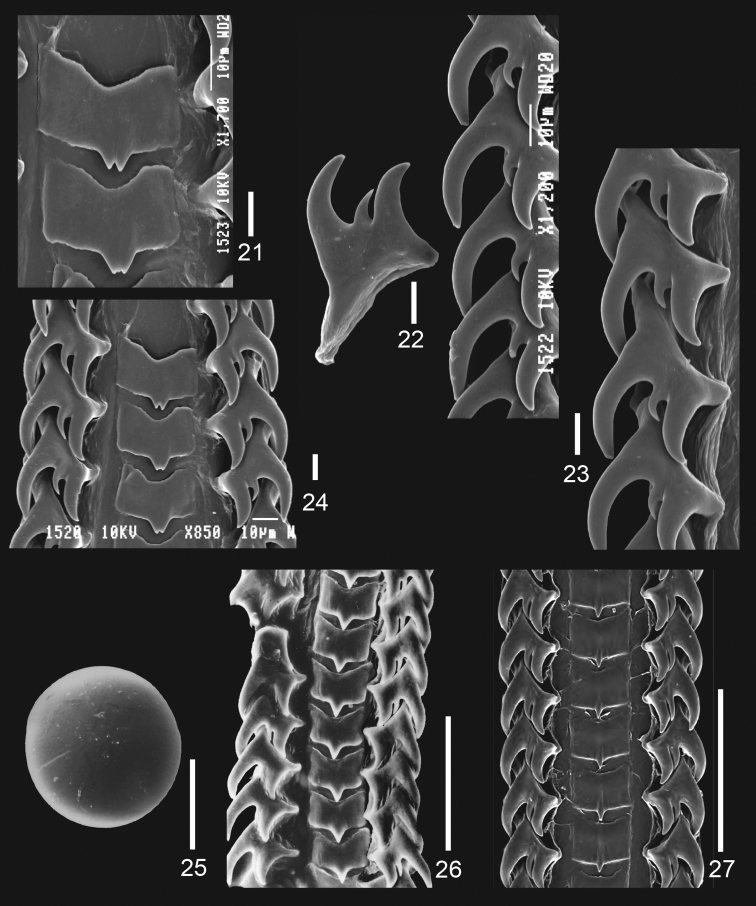
*Jerrybuccinum kantori* sp. n. **21–24** radula of paratype 1, scalebar: 10 micrometer **25** statocyst of paratype 1, scalebar: 100 micrometer **26** radula of *Jerrybuccinum explorator*, scalebar: 100 micrometer. **27** radula of *Jerrybuccinum malvinense*, scalebar: 100 micrometer, after Kantor & Pastorino, 2009: fig. 11.

##### Type locality.

Falkland Islands (Islas Malvinas), 52°00'S, 56°36'W, R/V ELTANIN, cruise 7, sta. 558, 14 Mar. 1963, 646–845 m.

##### Remarks.

The single shell that we studied is a slightly eroded and damaged empty shell collected off the Falkland Islands, but without exact locality data.

#### *Jerrybuccinum* species 1

Figures 17–18

**Remarks.** The single shell that we studied is a damaged empty shell collected off the Falkland Islands. This species differs from *Jerrybuccinum malvinense* by the broader shape, the bigger protoconch, the narrower axial ribs with broader interspaces, the finer spiral cords on the base and the larger size even though it is a subadult shell.

#### *Jerrybuccinum* species 2

Figures 19–20

**Remarks.** The single shell that we studied was collected north of the Falkland Islands. This species is characterised by having a weak axial sculpture consisting of narrow axial ribs on the spire whorls and a smooth body whorl; the absence of the spiral keel that delimits the shell base of typical *Jerrybuccinum*, but the presence of two slightly broader spiral cords. The protoconch is ornamented with fine spiral cords, a feature that is typical of the genus.

#### Jerrybuccinum explorator

(Fraussen & Sellanes, 2008) comb. n.

http://species-id.net/wiki/Jerrybuccinum_explorator

Figures 12–14 , 26

Kryptos explorator Fraussen & Sellanes, 2008: 102-104, figs 5–6, 16–25.

##### Type material.

Holotype in MNHNCL-5866, two paratypes in MNHNCL-5867-5868, two paratypes in KF-5180-5181 and a paratype in MNHN-9961.

##### Type locality.

South-central Chile, R/V Vidal Gormáz (SeepOx Cruise, AGT 6-7, 09/02/2006), CMSA, northwest of the Bay of Concepción, 36°20'97S, 73°44'86W, 850 m.

##### Remarks.

This species was tentatively placed in the genus *Kryptos* (Fraussen and Sellanes 2008) based on similarities in sculpture and radula and to prevent the description of a monotypic genus. The radula is identical to the radula of *Jerrybuccinum malvinense*. It is characterised by a monocuspid central tooth with quadrangular base and by tricuspid lateral teeth that have a broad base prolonged with a basal projection. The shape, the axial and spiral sculpture of the shell and the shape of the operculum are similar. We hereby assign this species to *Jerrybuccinum*.

#### Jerrybuccinum kantori sp. n.

http://zoobank.org/BCD550A7-F2E1-4584-93A9-8BAAA3E84665

http://species-id.net/wiki/Jerrybuccinum_kantori

Figures 1–11 , 21–25

##### Type material.

Holotype (MNHNCL-7589) (14.5 mm), Chile, northwest of the Bay of Concepción, R/V Melville, INSPIRE cruise, AGT 04, 36°23.595'S, 73°42.910'W, ~700 m deep, March 10, 2010, lv.

Paratype 1 (KF-5441) (12.1 mm), same locality as holotype, lv.

Paratype 2 (MNHNCL-7590) (15.7 mm), Chile, northwest of the Bay of Concepción, R/V Vidal Gormáz, ONR cruise, AGT 3.4, 36°10'S, 73°34'W, 521–613 m deep, October 2004, lv.

Paratypes 3–4 (KF-7019–7020) (13.6–11.6 mm), same locality as paratype 2, lv.

Paratypes 5–6 (MNHNCL-7591) (13.0–13.2 mm), Chile, northwest of the Bay of Concepción, R/V Vidal Gormáz, AIW cruise, 36°24.12'S, 73°36.44'W, 606 m deep, December 2003, lv.

Paratypes 7–8 (PS-150148) (12.5–10.5 mm) Chile, off El Quisco, R/V Melville, INSPIRE cruise, AGT 10, 33°23.378'S, 71°52.782'W, ~340 m deep, March 14, 2010, lv.

Paratype 9 (MNHNCL-7592) (9.5 mm), same locality as paratypes 7–8.

Paratype 10 (KF-5440) (8.8 mm), same locality as paratypes 7–8.

Paratypes 11–13 (CBUCN-003284) (8.1, 8.0, 6.2 mm juveniles), same locality as paratypes 7–8.

##### Type locality.

Chile, northwest of the Bay of Concepción, 36°23.595'S, 73°42.910'W, ~700 m deep.

##### Material examined.

Apart from the type material listed above 20 additional specimens (6.3–11.3 mm; 19 lv, 1 dd; JS) collected together with the paratypes 7 and 8 were studied.

Range and habitat: Only known from the type material and the specimens from off Concepción and off El Quisco. Most of the specimens of *Jerrybuccinum kantori* sp. n. collected so far were associated with fauna typical of methane seeps (vesicomyid, solemyid, lucinid, and thyasirid bivalves). However, the scarce knowledge of the bathyal SE Pacific malacofauna still keeps us from establishing this species as an obligatory dweller of seep environments or other reducing habitats.

##### Description.

Shell small for genus (up to 15.7 mm), thin but solid, semi-transparent, off-white. Shape broadly fusiform with high spire and moderately short siphonal canal.

Apex and protoconch eroded in all studied specimens. Remaining teleoconch whorls 6 (holotype) or 7 (paratype 3) in number, convex, adapical part slightly flattened, thereby accentuating a rather carinated shape. Suture distinct.

Upper teleoconch whorls with 5 or 6 fine spiral cords of unequal strength, separated by deep interspaces of equal width; slightly increasing in number. Penultimate whorl with 8–11 spiral cords, adapical spiral cords fine, abapical spiral cords slightly broader, interspaces of equal size or twice as wide. Body whorl with 21 or 22 fine spiral cords of unequal strength; 9 or 10 adapical spiral cords fine with moderately narrow interspaces; 2 or 3 interspaces situated along transition from whorl to base much broader; interspaces on base of unequal strength. Subadult shells with 2 more pronounced spiral cords visible. Siphonal canal with about 9 broad, flattened spiral cords separated by narrow interspaces.

Upper teleoconch whorls with 10 or 11 moderately narrow but pronounced, weakly bended axial ribs, slightly weaker near both sutures. Badly eroded ribs party or entirely decollate, forming a deep depression with sharp margins. Penultimate whorl with 13–16 weaker ribs on adapical half of body whorl. Base and upper border of subsutural slope smooth. Body whorl of adult specimens almost smooth. All whorls covered with fine, weakly curved incremental lines.

Aperture round; columella concave, smooth; callus thin, glossy. Outer lip thin, moderately sharp, laterally curved following the shape of the incremental lines. Siphonal canal narrow, rather short, open.

Operculum corneous, thin, elongate, concentric, nucleus situated near lower margin, almost terminal, forming a sharp tip. Colour pale brownish, with a slightly darker pattern forming a V-shaped mark that grows from the nucleus (Figs 8–11), juveniles with a thinner, more translucent operculum (Figs 10–11).

Periostracum greyish with a greenish shine, thin, smooth, well-adherent.

Radula (Figs 21–24) typical of genus: central tooth rather rectangular with concave base and 3, occasionally 1, short cusps; lateral teeth tricuspid with large outer cusp and small middle cusp.

Animal with a moderately large statocyst (Fig. 25), measuring more than 150 micrometres in diameter, found after dissolving the animal during radula preparation.

##### Comparison.

*Jerrybuccinum kantori* new species is characterised by having a moderately broad shape, a weakly carinated shape of the teleoconch whorls, axial ribs that are moderately broad and quite straight when compared to the other species of the genus and a small adult size.

*Jerrybuccinum explorator* from Chile differs by the more slender shape with higher spire, the more convex whorls, the numerous and narrower axial ribs that are also more twisted, the numerous and finer spiral cords, the browner periostracum and the larger adult size.

*Jerrybuccinum malvinense* (the type species of the genus) differs by the more slender shape with higher spire, the more convex whorls, the presence of an obvious, pronounced spiral cord ranging from whorl to base at the transition and the larger adult size.

##### Etymology.

*Jerrybuccinum kantori* new species is named to honour Yuri Kantor for his numerous important contributions to malacology.

## Supplementary Material

XML Treatment for Jerrybuccinum

XML Treatment for Jerrybuccinum malvinense

XML Treatment for Jerrybuccinum explorator

XML Treatment for Jerrybuccinum kantori
